# Apparent Kinetic Isotope Effects for Multi-Step Steady-State Reactions

**DOI:** 10.1021/acs.jpcb.5c00561

**Published:** 2025-03-27

**Authors:** Ian H. Williams

**Affiliations:** Department of Chemistry, University of Bath, Bath BA2 7AY, U.K.

## Abstract

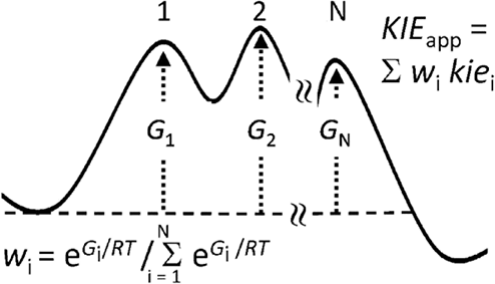

The apparent kinetic isotope effect (KIE) for a multistep steady-state reaction can be expressed simply as a sum of terms, one for each transition state (TS) in the serial sequence, each of which is the product of the KIE for an individual TS (with respect to a common reference state) and a weighting factor, which is the degree of kinetic significance for that TS. This requires knowledge of the relative Gibbs energies of the sequential TSs but not of any intermediates, and it involves a much simpler expression than the conventional method for analysis of KIEs for enzyme reactions. A numerical example is presented to illustrate how the same apparent KIE may result from numerous combinations of individual KIEs and weighting factors. It is proposed that computed apparent KIEs should be compared directly with experimentally observed KIEs rather than with derived intrinsic KIEs of possibly dubious validity. The results of DFT calculations for an S_N_1 nucleophilic displacement are presented to show how the apparent KIE varies, as the relative concentration of the nucleophilic species ranges from 0.1 to 10, between limiting values corresponding to either the first or second step being completely rate limiting.

## Introduction

**Scheme 1:**
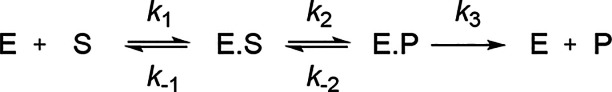
Minimal Enzyme Mechanism


The use of kinetic isotope effects (KIEs) as a tool for mechanistic elucidation of reactions across the breadth of chemistry and biochemistry has been amply demonstrated^1,2^ since their earliest application in the late 1940s.^3^ ln particular, understanding of KIEs for enzyme-catalyzed reactions under steady-state conditions has benefitted hugely from the seminal contributions of Northrop^4−6^ and of Cleland^7−9^ in developing methods for the determination, analysis, and interpretation of experimental observations. According to their treatment, an isotope effect on the observed value of *k*_cat_/*K*_m_ for a minimal enzyme mechanism (Scheme 1) involving only reversible substrate-binding (step 1) and chemical transformation (step 2), and irreversible product-release (step 3) may be expressed as eq 1, where the left-superscript notation indicates a tritium KIE resulting from substitution of this isotope in place of protium at a position involved in the chemical transformation of enzyme-bound substrate E.S to enzyme-bound product E.P. In principle, each step may be isotopically sensitive. ^T^*K*_1_ and ^T^*K*_2_ are equilibrium isotope effects for the first and second steps, and *C*_f_ (=*k*_2_/*k*_–1_) and *C*_r_ (=*k*_–2_/*k*_3_) are forward and reverse commitments to catalysis, respectively.^6^

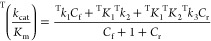
1

If it is assumed that only the chemical step 2 is isotopically sensitive, and that step 3 is irreversible, then eq 1 may be simplified to eq 2, and if *C*_f_ can be determined experimentally, then the ‘intrinsic’ KIE for step 2, ^T^*k*_2_, may be obtained easily from the observed, apparent value of ^T^(*k*_cat_/*K*_m_). The study by Singh and Schramm of multiple KIEs for the reaction catalyzed by 5′-methylthioadenosine nucleosidase (MTAN) is just one example (among many) that utilizes this approach.^10^
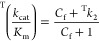
2

**Scheme 2:**
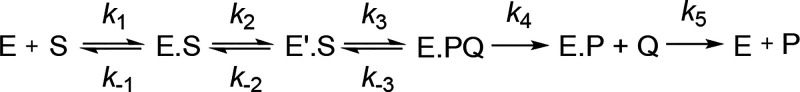
More-Complex Enzyme Mechanism


However, it is not always true that these simplifying assumptions are valid, and moreover, many enzymic reactions have more complex mechanisms involving multiple steps in series, leading to much more complex expressions for their KIEs. For example, an observed ^13^C KIE on *k*_cat_/*K*_m_ for the mechanism shown in Scheme 2 would be given by eq 3, where the left-superscript “1” indicates a parent (unsubstituted) isotopolog. In this case, obtaining an intrinsic KIE for an individual step would be very challenging.

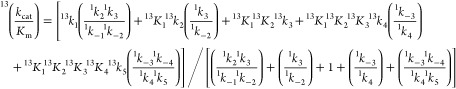
3

The complexity of eq 3, and of similar expressions that may be derived for other model mechanisms, is due to its focus on every serial intermediate in the mechanistic sequence and on the forward and reverse rate constants for each individual step. By analogy with Schowen’s terminology for descriptions of enzymic catalysis which (although being correct) omit or de-emphasize transition states in favor of intermediates,^11^ the Northrop-Cleland formulation of KIEs for enzymic reactions may be regarded as “canonical” Correspondingly, one may wonder if there is a “fundamentalist” formulation of enzymic KIEs that focuses instead on the serial transition states in a multistep sequence? As shown below, there is indeed an alternative and complementary approach that is particularly suited to the treatment of KIEs from computational simulations of enzymic reactivity and that focuses only on relative Gibbs energies of the significant transition states. It should be emphasized that both approaches are correct and can be shown to be equivalent, but the merit of the analysis presented below is its conceptual simplicity.

Most enzymic KIEs are determined by internal competition experiments, which yield only the isotope effect on *k*_cat_/*K*_m_ (and tritium KIEs can only be obtained by this means) and, moreover, Northrop’s method^6^ for deriving intrinsic KIEs requires that the observed isotope effect is upon *k*_cat_/*K*_m_; the following analysis therefore focuses upon this quantity. The Northrop–Cleland approach can also be applied to isotope effects on *k*_cat_, but it leads to even more complicated (and frightening!) expressions: the forward commitment *C*_f_ in eq 1 is replaced by a ratio *R*_f_/*E*_f_, where the numerator involves a sum of ratios of rate constants for every individual step and the denominator involves a sum of equilibrium constants.^6^ It will be seen that the approach described below applies equally well to isotope effects on *k*_cat_/*K*_m_ and *k*_cat_.

Since the kinetics of many multistep reactions which involve relatively high-energy reactive intermediates may be treated satisfactorily within the steady-state approximation, the considerations discussed below are much more widely applicable than to enzymic reactions only. However, it may be noted that the methodologies developed by enzymologists do not appear to have been generally adopted in other areas of chemistry, perhaps owing in part to differences in terminological usage.

## Gibbs-Energy Analysis of Steady-State Serial Reactions

Following Christiansen’s steady-state treatment,^12^ Noyes rigorously presented the kinetics for an unbranched sequence of reaction steps exhibiting uniform flux:^13^ the reciprocal net rate constant for forward reaction is the sum of reciprocals of rate constants for each individual step of the kinetically significant sequence, each being with respect to a common initial state. If the sequence is irreversible, the apparent rate constant *k*_app_ for overall reaction through a series of *n* steps is given by eq 4, where the index *i* may include as many steps as necessary, and where each *k*_*i*_ is now relative to the free substrate in (E + S) and *not* to its immediately preceding intermediate.
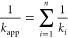
4

Within the transition state theory, each rate constant is related to a Gibbs energy difference between the common initial state (E + S) and the transition state (TS) for that step,^11^ as shown by eqs 5 and 6.
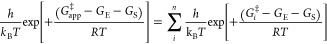
5
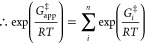
6
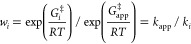
7

Several points may now be noted, as follows:

1. Since eqs 6 and 7 contain neither *G*_E_ nor *G*_S_, only relative TS energies *G*^‡^*_i_* are required.
2. The kinetic significance of each rate constant *k*_*i*_ is a weighting factor, *w*_*i*_ (eq 7) which measures the fractional degree to which that rate constant limits the overall rate; the sum of all the *w*_*i*_ is unity.^14,15^
3. Whereas the TS corresponding to each *k*_*i*_ is a real entity, the TS corresponding to *k*_app_ is an imaginary entity: a virtual TS.^11^
4. The Gibbs-energy terms in eqs 5–7 are all preceded by a *plus* sign, in contrast to a *minus* sign in the analogous expressions for the kinetics of reaction steps in parallel, for which *k*_app_ = Σ*k*_*i*_ with the contribution of each TS to the virtual TS (corresponding to *k*_app_) being its Boltzmann weighting. For a sequence of real TSs in series, the apparent Gibbs energy of activation Δ^‡^*G*_app_ is *greater* than the value of Δ^‡^*G*_*i*_ for any individual step (relative to the common initial state) and the TS with the highest Gibbs energy has the greatest kinetic significance. In contrast, for a wall of real TSs in parallel, Δ^‡^*G*_app_ is *less* than the value of Δ^‡^*G*_*i*_ for any of the contributing alternate reaction paths and the TS with the lowest Gibbs energy has the greatest kinetic significance.

Similar findings have been reported by others.^16−19^

## KIEs for Multi-Step Enzymic Reactions in the Steady State

It can be shown easily that the weighting factors *w*_*i*_ for the minimal enzyme mechanism (Scheme 1) are related (eq 8) to the commitments to catalysis, *C*_r_ and *C*_f_, and that the (tritium) isotope effect on *k*_cat_/*K*_m_ is the weighted arithmetic mean of KIEs on the *n* = 3 individual steps with respect to a common reactant state (eq 9).
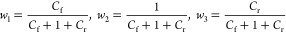
8
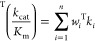
9

**Figure 1:**
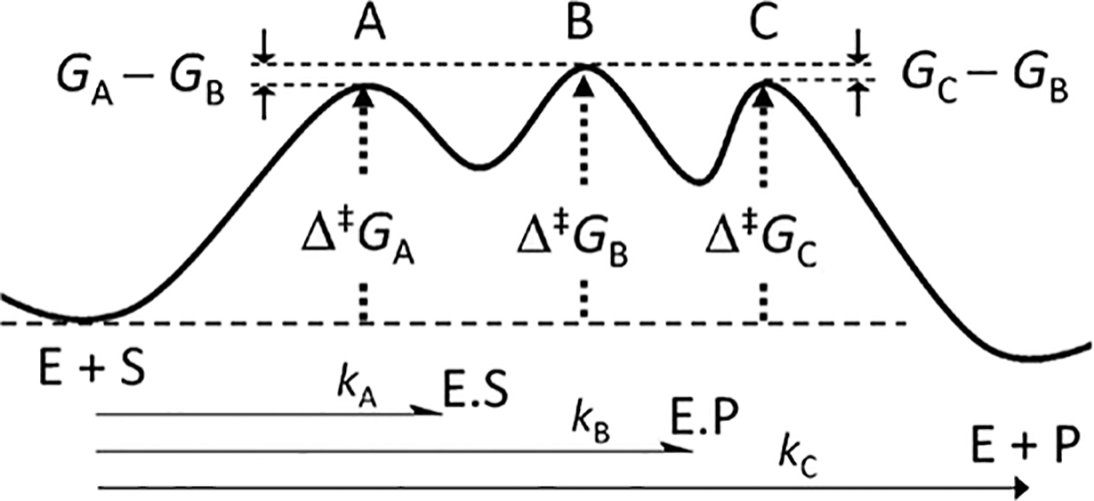
Gibbs energy profile corresponding to a minimal enzyme mechanism.

Combining eqs 7 and 8 allows the forward and reverse commitment factors for the minimal enzyme mechanism to be expressed in terms of Gibbs energies of the three TSs: A, B, and C, namely, *G*_A_, *G*_B_, and *G*_C_ (eq 10). Figure 1 shows the Gibbs energies of activation Δ^‡^*G*_A_, Δ^‡^*G*_B_, and Δ^‡^*G*_C_ relative to E + S and the corresponding rate constants *k*_A_, *k*_B_, and *k*_C_. Equation 10 involves only Gibbs energy differences between the TSs; as drawn in Figure 1, B is higher in energy than either A or C, and so these differences are negative quantities, leading to *C*_r_ and *C*_f_ both being less than unity.
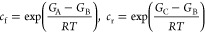
10

This provides a clear picture of the physical significance of *C*_f_ and of *C*_r_ as being akin to equilibrium constants for partitioning between TS B and either TS A (forward) or TS C (reverse). If either *G*_A_ or *G*_C_ is equal to *G*_B_ then the corresponding commitment is unity, but if *G*_B_ is very much greater than either *G*_A_ or *G*_C_ then it tends to zero. Equation 9 can be generalized for any number of steps in series, e.g., where *n* = 5 for the more complex mechanism of Scheme 2, and similar findings have been reported by Ruszczycky.^20,21^ However, to treat more steps in a serial mechanism using the ‘canonical’ Northrop-Cleland approach requires a *C*_f_ and a *C*_r_ to be defined for each additional step, and the formulation becomes very messy. In contrast, the ‘fundamentalist’ approach requires only the Gibbs energy for each additional TS, which is much simpler. Admittedly, these TS properties are not easily obtainable experimentally, and so, the Northrop-Cleland method continues to be employed, despite caveats regarding the validity of the simplifying assumptions it requires.

## Computational Simulation of KIEs for Multi-Step Steady-State Reactions

Modern methods of computational simulation now provide realistic tools, complementary to experiments, for the investigation of mechanisms^22^ and KIEs^23^ for enzymatic reactions. Since suitable QM/MM methods, for example, allow for TSs and intermediates to be located along a reaction pathway, their relative free energies to be determined, and their isotopic sensitivities to be evaluated, it is now reasonably straightforward to compute both the weighting factors *w*_*i*_ and KIEs associated with individual kinetically significant TSs for any desired isotopic substitution, by means of eq 11. Here, tritium substitution is used again as an example; *f*_s_ and *f*_*i*_ indicate isotopic partition-function ratios (IPFRs) for the free substrate in aqueous solution and for the *i*th enzymatic TS, respectively, and the brackets ⟨···⟩ denote averaging over numerous thermally accessible configurations.
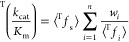
11

The use of eq 11 allows computed KIEs to be compared directly to an observed isotope effect on *k*_cat_/*K*_m_ for multiple isotopic substitutions. Although computational simulations may also provide KIEs and EIEs for individual elementary steps, comparison with experimentally derived intrinsic KIEs may not be desirable if there is any doubt associated with the validity of the latter. It is important to note that, within the “fundamentalist” approach, IPFRs need to be evaluated only for each TS and not for any intermediate species.

It follows from the last point that an isotope effect upon *k*_cat_ can be expressed by a simple modification of eq 11 in which the IPFR for the free substrate is replaced by that for the enzyme-bound substrate, as in eq 12.
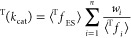
12

## Numerical Example for an Enzymic Reaction

Intrinsic KIEs determined experimentally by Singh and Schramm for the *S. pneumoniae* MTAN-catalyzed hydrolysis of 5′-methylthioadenosine (MTA) suggested an S_N_1-like mechanism with a ribooxacarbenium ion intermediate.^10^ Their derivation assumed that there was no equilibrium isotope effect upon substrate binding and a reverse commitment equal to unity; a forward commitment was measured experimentally. Thence a value for the intrinsic KIE for tritium substitution at the anomeric carbon (a 2° α-T KIE) was obtained by application of eq 2, along with intrinsic KIEs for other isotopic substitutions. However, preliminary results from a QM/MM computational modeling study by Glancy^24^ for this system indicate that each step may be isotopically sensitive; this work will be presented in full elsewhere in due course. However, in the present author’s mind, this finding not only casts some doubt upon the reliability of the reported intrinsic KIEs but also prompts the Gibbs-energy analysis outlined above, with a view to proposing that computed apparent KIEs should be compared directly to observed KIEs rather than to derived intrinsic KIEs. To this end, it is instructive to consider a purely numerical exercise with eq 13 to illustrate how an observed 2° α-T KIE on *k*_cat_/*K*_m_ for a minimal enzyme mechanism (Scheme 1 and Figure 1) may vary with changing values of Gibbs energies, weighting factors, and IPFRs for each of the TSs involved.
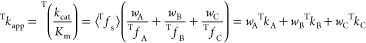
13

It is important to note that the KIEs ^T^*k*_A_, ^T^*k*_B_, and ^T^*k*_C_ are not intrinsic KIEs as the reference state for them all is the free substrate in solution, not the intermediate that immediately precedes each TS. The value of *G*_A_ – *G*_B_ = −5.44 kJ mol^–1^ used for all cases considered in Table 1 corresponds to the forward commitment *C*_f_ = 0.121 as determined experimentally for MTAN-catalyzed MTA hydrolysis, and the target value of ^T^*k*_app_ = 1.210 is the experimentally observed value for this in reaction.^10^

**Table 1:** Numerical Simulation of Isotope Effects for the Minimal Enzyme Mechanism (Scheme 1 and Figure 1) at 310 K^a^

case	*G*_A_ – *G*_B_	*G*_C_ – *G*_B_	*C*_f_	*C*_r_	*w*_A_	*w*_B_	*w*_C_	^T^*k*_A_	^T^*k*_B_	^T^*k*_C_	^T^*k*_app_
1a	–5.44	–13	0.121	0.006	0.107	0.887	0.006	1.000	1.235	1.279	1.210
1b	–5.44	–10	0.121	0.021	0.106	0.876	0.018	1.000	1.235	1.231	1.210
1c	–5.44	–7	0.121	0.066	0.102	0.842	0.056	1.000	1.235	1.217	1.210
2a	–5.44	–10	0.121	0.021	0.106	0.876	0.018	1.050	1.228	1.300	1.210
2b	–5.44	–5	0.121	0.144	0.096	0.791	0.114	1.050	1.216	1.300	1.210
2c	–5.44	0	0.121	1.000	0.057	0.471	0.471	1.050	1.139	1.300	1.210
3	–5.44	–10	0.121	0.021	0.106	0.876	0.018	1.050	1.235	0.950	1.210

a Gibbs energy differences are in kJ mol^–1^.

Cases 1a, 1b, and 1c all assume that there is no KIE on the substrate binding step via TS A, i.e., ^T^*k*_A_ = 1, and differ only in the magnitude of the Gibbs energy difference *G*_C_ – *G*_B_, with TS B being of higher energy than both A and C. Experimentally, the reaction was found to be irreversible under initial rate conditions and therefore it was assumed that *C*_r_ = 0. Table 1 shows *C*_r_ to be very small but not zero: as *G*_C_ – *G*_B_ becomes less negative, both *C*_r_ and *w*_C_ increase in value. As expected, B is the most kinetically significant TS, having the highest Gibbs energy and the largest weighting factor, *w*_B_. For the sake of this demonstration, ^T^*k*_B_ = 1.235 (which happens to be the same value as the intrinsic KIE in ref,^10^ but the same target value for ^T^*k*_app_ can be achieved by choosing an appropriate value for the parameter ^T^*k*_C_ within a plausible range for this KIE. Cases 2a, 2b, and 2c use a fixed (and plausible) value for ^T^*k*_C_ but treat ^T^*k*_B_ as a variable parameter; also, a small normal value for ^T^*k*_A_ is assumed. As *G*_C_ – *G*_B_ becomes less negative, ^T^*k*_app_ = 1.210 is achieved for decreasing magnitudes of ^T^*k*_B_. Finally, case 3 considers the possibility of a small inverse value for ^T^*k*_C_, a small normal value for ^T^*k*_A_, together with the same negative *G*_C_ – *G*_B_ value as case 1b, and again yields the same value for ^T^*k*_app_.

Of course, these cases could be extended, but this small selection is sufficient to make the point that there are potentially many ways by which a particular value of the apparent KIE can arise, depending on the weighting factors and isotopic sensitivities of the TSs involved. Computational modeling with QM/MM methodologies can usefully be employed to locate and characterize the kinetically significant TSs, to determine their relative Gibbs energies and IPFRs, and thereby to evaluate the apparent KIE for any given isotopic substitution for comparison with the experimentally observed KIE.

## Computational Simulation of KIEs for Stepwise Nucleophilic Displacement in Solution

**Scheme 3:**
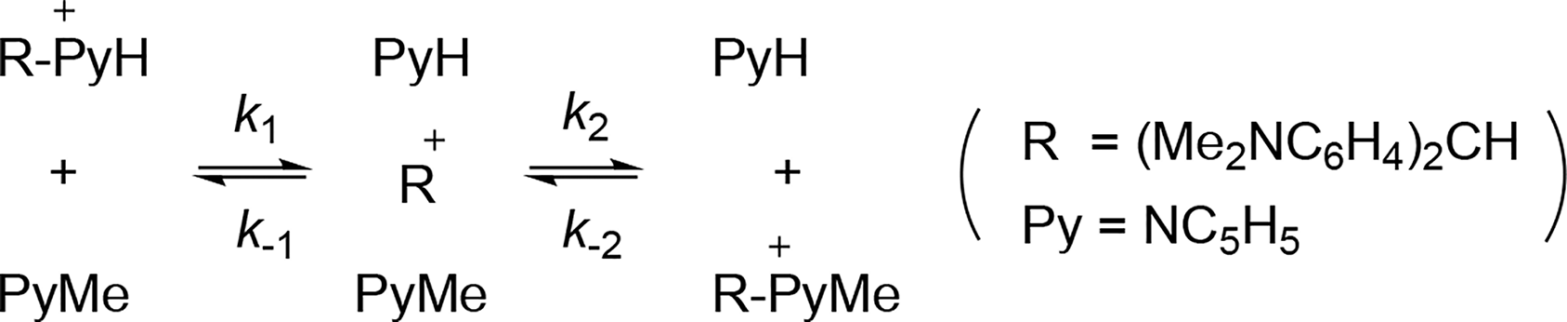
S_N_1 Nucleophilic Displacement

We recently discussed apparent Gibbs energies of activation for reacting systems with multiple reactant-state (RS) and TS conformers, using the example of solvolysis of a 4,4′-disubstituted benzhydrylpyridinium salt by an S_N_1 mechanism to illustrate correct and incorrect ways of using computed energies for the reactant structures and transition structures as obtained from a published source.^25^ It is convenient to use the same source now to provide DFT-optimized (M06-2X/6-311+G(2d,p)/PCM = EtOH) structures for the species involved in Scheme 3, where *R* represents the bis(4,4′-dimethylamino)benzhydryl group and Py represents the pyridinyl group. The vibrational Hessian has now been computed for each species, at the same level of theory, from which harmonic vibrational frequencies have been obtained with both protium and deuterium attached to the benzhydryl α-carbon atom (See the Supporting Information for details.)

Provided that the Gibbs energy for heterolysis of either substituted benzhydrylpyridinium cation is sufficiently endoergic so that the reactive intermediate R^+^ is high in energy relative to reactants or products, the steady state approximation may be applied, since *k*_2_ ≫ *k*_1_ and *k*_–1_ ≫ *k*_–2_ so that R^+^ disappears quickly once it is formed.^26^ The 2° α-D KIE for the overall reaction in the forward direction is hence given by eq 13, where A and B now refer to the TSs for the first and second steps in Scheme 3, and the *k*_A_ and *k*_B_ values are both with respect to a common reactant state.
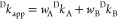
14

By default, the computed Gibbs energies used to obtain the weighting factors *w*_A_ and *w*_B_ are standard molar quantities, implying that nucleofuge PyH and nucleophile PyMe would be present in equimolar quantities. However, in order to replicate probable experimental conditions required to drive the overall equilibrium toward products, it would be necessary to use an excess of the PyMe, and so its Gibbs energy must be corrected for its increased concentration relative to that of RPyH^+^: a term equal to -*RT*ln([PyMe]/[RPyH^+^]) must be added to the Gibbs energy of TS B before *w*_A_ and *w*_B_ are calculated. The standard molar Gibbs energy of the reaction for Scheme 3 is exoergic, and the energy of TS B is also lower than that of TS A. Increasing the relative concentration of PyMe increases the ratio [PyMe]/[RPyH^+^] and leads to an even more negative value of *G*_B_ – *G*_A_, whereas decreasing the relative concentration of PyMe serves to raise the Gibbs energy of the TS for the second step in Scheme 3.

**Figure 2:**
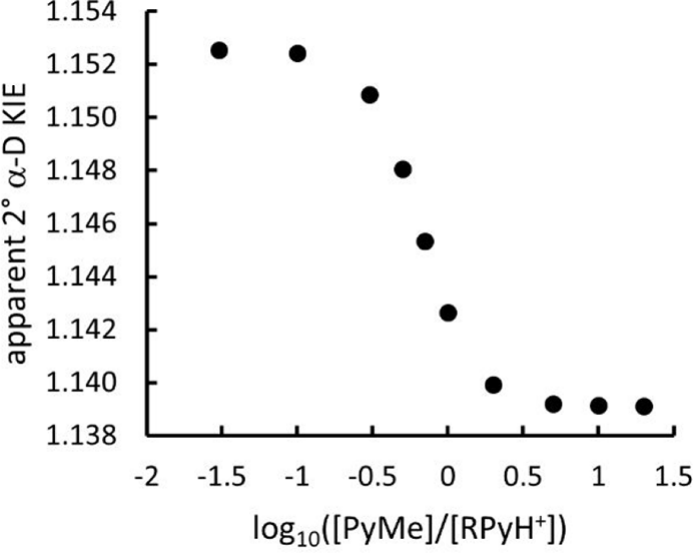
Variation of the apparent 2° α-D KIE for the reaction of Scheme 3 as a function of the relative concentration of the PyMe nucleophile to the RPyH^+^ substrate at 298 K.

The abscissa in the plot shown in Figure 2 is displayed as the decadic, rather than the natural, logarithm of the ratio [PyMe]/[RPyH^+^] for ease of interpretation. As this quantity becomes more positive, the value of *w*_A_ approaches unity and the apparent KIE tends toward the limit (at 298 K) of 1.139 for completely rate-limiting heterolysis, with the departure of the PyH nucleofuge from the intermediate benzhydryl cation. Conversely, as log([PyMe]/[RPyH^+^]) becomes more negative, the value of *w*_B_ approaches unity and the apparent KIE tends toward the limit 1.153 for completely rate-limiting addition of the PyMe nucleophile to the intermediate R^+^. For equimolar concentrations of PyMe and RPyH^+^, the weighting factors for the two TSs are *w*_A_ = 0.73 and *w*_B_ = 0.27, and the value of ^D^*k*_app_ is therefore a weighted average of the two limiting values. The key point to note is that the apparent KIE which would be observed experimentally varies between the limiting values as the relative nucleophile concentration changes over 2 orders of magnitude, i.e., 0.1 < [PyMe]/[RPyH^+^] < 10.

## Concluding Remarks

A classic text on chemical kinetics sought explicitly to bring together both the macroscopic and microscopic aspects of the subject;^27^ today a similar intent would certainly need also to include the role of computational modeling. A continuing challenge to those who practice in interdisciplinary areas is to communicate effectively across the language barrier that too often separates different communities within chemistry and its allied subjects.^28^ Arguably, this issue has unhelpfully restricted interaction between enzymologists and physical chemists over many years, not least regarding the application of KIEs for the elucidation of complex reaction mechanisms. Indeed, some important past contributions^11,13^ have become buried deep in the literature and (almost) completely lost from view. Meanwhile, the rise of computational chemistry as a powerful tool, complementary to experiment, has sometimes failed to appreciate all that is required to relate computed results meaningfully to experimental observations. Perhaps a spirit of humility is necessary to acknowledge that neither experiment alone nor computation alone can provide all the answers but that together lies a way to make progress.

The conventional approach for the analysis of KIEs for steady-state enzyme reactions^4−9^ has been remarkably useful, but it is limited by the complexity of the kinetic expressions involved when based upon individual rate constants for every separate elementary step and by the need to make simplifying assumptions that may not be valid. Is the focus on the role of intermediate species a consequence of writing reaction schemes in terms of equilibrium species (reactants, intermediates, and products) rather than in terms of the transition states that interconnect them? There is, of course, good reason to focus attention on species that are more readily amenable to experimental observation, but transition states are elusive—by definition! On the other hand, while searching for saddle points on multidimensional potential energy hypersurfaces is often not an easy task, in principle a transition structure is as amenable to computational characterization as any other structure lying at an energy minimum. Moreover, with care, it is now possible to perform simulations yielding relative Gibbs energies and other important properties, such as IPFRs, that allow the gap between the microscopic and macroscopic domains to be bridged.

A key motivation of this work is to offer a complementary perspective on the kinetics of complex mechanisms, in terms of Gibbs energy differences, which provides considerable simplification and clarity. However, because of its focus on transition states, it presupposes that reliable estimates of TS properties are available. It remains a challenge to establish robust and accurate computational methodologies to achieve this, along with the expertise to apply them appropriately to complex chemical and biochemical processes without oversimplification. Notwithstanding, and without underestimating these difficulties, it is now proposed that the goal of computational simulations of KIEs should be to evaluate the apparent KIE corresponding directly to experimental observation rather than to derive intrinsic KIEs of possibly dubious validity.

A suggested computational protocol for a putative reaction mechanism would be as follows: (1) Determining the Gibbs energy and the IPFR for the reactant state and each transition state (with appropriate averaging over an adequate sampling of thermally accessible configurations). (2) Either eq 11 or eq 12 is applied to obtain the apparent KIE on, respectively, *k*_cat_/*K*_m_ or *k*_cat_. (3) The weighted-average computed KIE is directly compared with the experimentally observed KIE to assess the plausibility of the proposed mechanism. If desired, the KIE on each and every individual step of a putative mechanism may also be computed from the IPFRs for a particular TS and its immediately preceding intermediate; any one of these may be compared to an experimentally derived intrinsic KIE, if such is available, subject to the caveat previously noted regarding reliability.

Finally, although textbooks may describe the limiting cases of serial multistep reactions in the steady state, where either one TS or another becomes completely rate-limiting (e.g., ref (29)), this author is unaware of published descriptions for such systems that consider how KIEs should be treated when two or more TSs are each partially rate-limiting and when their kinetic significances may vary with experimental conditions.

## Supporting Information Available

Computed Gibbs energies, and IPFRs and derived quantities for structures involved in the stepwise nucleophilic displacement in solution (PDF)

## References

[ref1] Isotope Effects in Chemistry and Biology; Kohen A.; Limbach H.-H., Eds.; Taylor and Francis: New York, 2006.

[ref2] Isotope Effects in the Chemical, Geological, and Bio Sciences; Wolfsberg M.; Van Hook W. A.; Paneth P.; Rebelo L. P. N.; Springer: Dordrecht, 2010.

[ref3] Melander L. Mechanism of nitration of the aromatic nucleus. Nature 1949, 163, 599–599. 10.1038/163599a0. 18117129

[ref4] Northrop D. B. Steady-state analysis of kinetic isotope effects in enzymic reactions. Biochemistry 1975, 14, 2644–2651. 10.1021/bi00683a013. 1148173

[ref5] Northrop D. B. Determining the Absolute Magnitude of Hydrogen Isotope Effects. In Isotope Effects on Enzyme-Catalyzed Reactions. Cleland W. W.; O’Leary M. H.; Northrop D. B., Eds., University Park Press: Baltimore, 1977.

[ref6] Northrop D. B. Intrinsic isotope effects in enzyme-catalyzed reactions. In Enzyme Mechanism from Isotope Effects, Cook P. F., Ed., CRC Press: Boca Raton, 1991.

[ref7] Cleland W. W. Partition analysis and the concept of net rate constants as tools in enzyme kinetics. Biochemistry 1975, 14, 3220–3224. 10.1021/bi00685a029. 1148201

[ref8] Cleland W. W. The use of isotope effects to elucidate enzyme mechanism. CRC Crit. Rev. Biochem. 1982, 13, 385–428. 10.3109/10409238209108715. 6759038

[ref9] Cleland W. W. The use of isotope effects in the detailed analysis of catalytic mechanisms of enzymes. Bioorg. Chem. 1987, 15, 283–302. 10.1016/0045-2068(87)90026-5.

[ref10] Singh V.; Schramm V. L. Transition-state analysis of *S. pneumoniae* 5′-methylthioadenosine nucleosidase. J. Am. Chem. Soc. 2007, 129, 2783–2795. 10.1021/ja065082r. 17298059 PMC2522316

[ref11] Schowen R. L. Catalytic Power and Transition-State Stabilization. In Transition States of Biochemical Processes, Gandour R. D.; Schowen R. L., Eds., Plenum Press: New York, 1978, 77–114.

[ref12] Christiansen J. A. Einige Bemerkungen zur Anwendung der Bodenstein sehen Methode der stationären Konzentrationen der Zwischenstoffe in der Reaktionskinetik. Zeit. Phys. Chem. 1935, 28B, 303–310. 10.1515/zpch-1935-2828.

[ref13] Noyes R. M. Kinetic treatment of consecutive processes. Prog. React. Kinet. 1964, 2, 339–362.

[ref14] Alvarez F. J.; Ermer J.; Hübner G.; Schellenberger A.; Schowen R. L. Catalytic power of pyruvate decarboxylase. rate-limiting events and microscopic rate constants from primary carbon and secondary hydrogen isotope effects. J. Am. Chem. Soc. 1991, 113, 8402–8409. 10.1021/ja00022a030.

[ref15] Stein R. L. Analysis of kinetic isotope effects on complex reactions utilizing the concept of the virtual transition state. J. Org. Chem. 1981, 46, 3328–3330. 10.1021/jo00329a036.

[ref16] Ray W. J. Rate-limiting step: a quantitative definition. application to steady-state enzymic reactions. Biochemistry 1983, 22, 4625–4637. 10.1021/bi00289a003. 6626520

[ref17] Yomo T.; Yamano T.; Yamamoto K.; Urabe I. General equation of steady state enzyme kinetics using net rate constants and its application to the kinetic analysis of catalase reaction. J. Theor. Biol. 1977, 188, 301–312. 10.1006/jtbi.1997.0474. 9344734

[ref18] Dale H. J. A.; Leach A. G.; Lloyd-Jones G. C. Heavy-atom kinetic isotope effects: primary interest or zero point?. J. Am. Chem. Soc. 2021, 143, 21079–21099. 10.1021/jacs.1c07351. 34870970

[ref19] Park C. Visual interpretation of the meaning of *k*_cat_/*K*_m_ in enzyme kinetics. J. Chem. Educ. 2022, 99, 2556–2562. 10.1021/acs.jchemed.1c01268.

[ref20] Ruszczycky M. W.; Anderson V. E. Interpretation of *V*/*K* isotope effects for enzymatic reactions exhibiting multiple isotopically sensitive steps. J. Theor. Biol. 2006, 243, 328–342. 10.1016/j.jtbi.2006.06.022. 16914160

[ref21] Ruszczycky M. W.; Liu H.-W. Theory and application of the relationship between steady-state isotope effects on enzyme intermediate concentrations and net rate constants. Method. Enzymol. 2017, 596, 459–499. 10.1016/bs.mie.2017.07.022. PMC5837895 28911781

[ref22] Ranaghan K. E.; Mulholland A. J. QM/MM methods for simulating enzyme reactions. In Simulating Enzyme Reactivity: Computational Methods in Enzyme Catalysis, Tuñón I.; Moliner V., Eds., Royal Society of Chemistry: Cambridge, U.K., 2017.

[ref23] Wilson P. B.; Williams I. H. Kinetic isotope effects. In Simulating Enzyme Reactivity: Computational Methods in Enzyme Catalysis, Tuñón I.; Moliner V., Eds., Royal Society of Chemistry: Cambridge, U.K., 2017.

[ref24] Glancy J. H. M. Kinetic isotope effects as transition-state probes for glycosidic reactions. PhD thesis, University of Bath, 2022.

[ref25] Matić M.; Denegri B. DFT-PCM study on solvolytic behaviour of *N*-alkyl-*X*-pyridinium ions. ChemistrySelect 2021, 6, 2410–2423. 10.1002/slct.202004231.

[ref26] Volk L.; Richardson W.; Lau K. H.; Hall M.; Lin S. H. Steady state and equilibrium approximations in reaction kinetics. J. Chem. Educ. 1977, 54, 95–97. 10.1021/ed054p95.

[ref27] Steinfeld J. I.; Francisco J. S.; Hase W. L. Chemical Kinetics and Dynamics; Prentice Hall: Englewood Cliffs, 1989.

[ref28] Williams I. H. Physical organic chemistry in the 21st century: a first-quarter progress report. Chem. Int. 2022, 44, 10–13. 10.1515/ci-2022-0203.

[ref29] Maskill H. The Physical Basis of Organic Chemistry; Oxford University Press: Oxford, 1985.

