# Supplementary material for: Apparent Kinetic Isotope Effects for Multi-Step Steady-State Reactions
Source: J Phys Chem B. 2025 Mar 27;129(14):3604–9. doi: 10.1021/acs.jpcb.5c00561 (PMC11995368; doi:10.1021/acs.jpcb.5c00561)
Supplement: Supplementary file 1 — jp5c00561_si_001.pdf [file jp5c00561_si_001.pdf]

Apparent Kinetic Isotope Effects for Multi-Step Steady-State Reactions

Ian H. Williams

Department of Chemistry, University of Bath, Bath BA2 7AY, United Kingdom

Email: i.h.williams@bath.ac.uk

Supporting Information

COMPUTATIONAL SIMULATION OF KIEs FOR A STEPWISE NUCLEOPHILIC DISPLACEMENT IN SOLUTION

Computational methods

Cartesian coordinates for each species were taken directly from the Supporting Information of the paper by Matić and Denegri[1] on heterolysis of *N*-alkyl-*X*-pyridinium ions). A frequency calculation was performed for each already-optimized structure using the Gaussian16 program[2] with the following keywords.

M062X/6-311+G(2d,p) scrf=(solvent=ethanol) int=superfine freq punch=(coord,derivatives)

Mass-weighting and diagonalization of the computed Hessian written to the Gaussian "punch" file yielded 3*N* non-zero harmonic vibrational frequencies (for an *N*-atomic molecule). These frequencies, together with the atomic masses and Cartesian coordinates, were used within the standard harmonic-oscillator, rigid-rotor, ideal-gas approximations to obtain molecular partition functions *q* as products of translational, rotational, and vibrational (including zero-point energy) factors, from which isotopic partition-function ratios ($f = q_{\text{heavy}}/q_{\text{light}}$) were obtained by means of the LIPFR program from the SULISO suite of utilities,[3] using eqs 1- 5. Projecting out the spurious translational and rotational contributions from the computed Hessian (by means of the CAMVIB program from the SULISO suite) yielded six zero frequencies and 3*N* – 6 non-zero frequencies corresponding to pure vibrational modes.[4,5] No scaling was applied to any of the calculated vibrational frequencies.

$$f = \frac{q_{\text{heavy}}}{q_{\text{light}}} = \text{IM} \times \text{EX} \times \text{ZP} \times \text{TV} \tag{1}$$

$$\text{IM} = \left(\prod_j^N \frac{m_j}{m_j'}\right)^{3/2} \tag{2}$$

$$\text{EX} = \prod_{i=2}^{3N-6} \frac{1-\exp(-u_i)}{1-\exp(-u_i')} \tag{3}$$

$$\text{ZP} = \prod_{i=2}^{3N-6} \frac{\exp(½u_i)}{\exp(½u_i')} \tag{4}$$

$$\text{TV} = \frac{\omega_1' \sin(½u_\ddagger)}{\omega_1 \sin(½u_\ddagger')} \tag{5}$$

Here $u_i = hc\omega_i/k_BT$, ω_i is an unscaled harmonic frequency (as a wavenumber/cm^{-1}), *T* is the absolute temperature, *h* is Planck's constant, *c* is the velocity of light, and k_B is Boltzmann's constant; quantities pertaining to the heavy isotopolog are denoted by a prime. IM is the isotopic mass factor; EX is the excitational factor due to the population of vibrational energy levels with v > 0, and ZP is the zero-point energy factor. TV is the leading term of the Bell model for tunneling through an inverted parabolic barrier.[6,7] The overall expression for *f* (eq 1) includes a quantum correction to the partition function not only for each separable vibrational mode with a real frequency (i.e. zero-point energy) but also for motion in the transition vector with its imaginary frequency $\omega_\ddagger$. All three factors EX, ZP and TV are quantum corrections to ratios of classical partition functions, not only the one associated with tunneling.

Computed Gibbs energies, IPFRs and derived quantities

The quantity G_{total} in the following Tables is the 'sum of electronic and thermal enthalpies' displayed in the output from G16 for the default temperature T = 298.15 K. Deuteriated species are displayed as α-^{2}H and protiated species as α-^{1}H.

Reactant conformers

Note: RT at 298.15 = 2.47897 kJ mol^{-1}

Table S1. M062X/6-311+G(2d,p)/PCM=EtOH Gibbs energies, mole fractions, and α-^{2}H IPFRs (f) for conformers of the pyridinium ion at 298 K.

conformer	$(Me_2NC_6H_4)_2CH$-PyH						
	G_{total} α-^{1}H	*Boltzmann population* n_B	*Mole fraction* x	G_{total} α-^{2}H	*Boltzmann population* n_B	*Mole fraction* x	f α-^{2}H
1	-1017.545686	0.781319	0.238246	-1017.549326	0.787133	0.238400	47.445078
2	-1017.545572	0.692456	0.211149	-1017.549218	0.702056	0.212633	47.781510
3	-1017.545919	1.000000	0.304928	-1017.549552	1.000000	0.302871	47.121532
4	-1017.545715	0.805688	0.245677	-1017.549356	0.812544	0.246096	47.484568
weighted average	-1017.545740			-1017.549379			47.427159

Table S2. M062X/6-311+G(2d,p)/PCM=EtOH Gibbs energies, mole fractions, and α-^{2}H IPFRs (f) for conformers of the 4-methylpyridinium ion at 298 K.

conformer	$(Me_2NC_6H_4)_2CH$-PyMe						
	G_{total} α-^{1}H	*Boltzmann population* n_B	*Mole fraction* x	G_{total} α-^{2}H	*Boltzmann population* n_B	*Mole fraction* x	f α-^{2}H
1	-1056.833717	0.454283	0.156860	-1056.837350	0.454283	0.156768	47.115441
2	-1056.833724	0.457663	0.158027	-1056.837358	0.458148	0.158102	47.118811
3	-1056.834462	1.000000	0.345292	-1056.838095	1.000000	0.345089	47.069524
4	-1056.834138	0.709531	0.244995	-1056.837772	0.710283	0.245111	47.165473
5	-1056.832395	0.112009	0.038676	-1056.836029	0.112128	0.038694	47.118486
6	-1056.832747	0.162615	0.056150	-1056.836382	0.162960	0.056236	47.127338
weighted average	-1056.833973			-1056.837606			47.113162

Transition structures

Table S3. M062X/6-311+G(2d,p)/PCM=EtOH Gibbs energies for transition structures A and B at 298 K.

$(Me_2NC_6H_4)_2CH$-PyH		$(Me_2NC_6H_4)_2CH$-PyMe	
α-^{1}H	α-^{2}H	α-^{1}H	α-^{2}H
-1017.522317	-1017.525834	-1056.808936	-1056.812442

Other structures

Table S4. M062X/6-311+G(2d,p)/PCM=EtOH Gibbs energies for intermediate cation and pyridine species at 298 K.

$(Me_2NC_6H_4)_2CH^+$	PyH	PyMe
-769.3539698	-248.189575	-287.4752313

Gibbs energies of reaction

$(Me_2NC_6H_4)_2CH$-PyH $\rightarrow$ $(Me_2NC_6H_4)_2CH^+$ + PyH $\quad (\Delta_{rxn}G/RT) = 2.325$

$(Me_2NC_6H_4)_2CH$-PyMe $\rightarrow$ $(Me_2NC_6H_4)_2CH^+$ + PyMe $\quad (\Delta_{rxn}G/RT) = 5.054$

Gibbs energies of activation

$(Me_2NC_6H_4)_2CH$-PyH $\rightarrow$ $[(Me_2NC_6H_4)_2CH\text{-PyH}]^\ddagger$ $\quad (\Delta^\ddagger G/RT) = 24.81$

$(Me_2NC_6H_4)_2CH$-PyMe $\rightarrow$ $[(Me_2NC_6H_4)_2CH\text{-PyMe}]^\ddagger$ $\quad (\Delta^\ddagger G/RT) = 26.52$

$(Me_2NC_6H_4)_2CH^+$ + PyH $\rightarrow$ $[(Me_2NC_6H_4)_2CH\text{-PyH}]^\ddagger$ $\quad (\Delta_{int}G/RT) = 22.48$

$(Me_2NC_6H_4)_2CH^+$ + PyH $\rightarrow$ $[(Me_2NC_6H_4)_2CH\text{-PyH}]^\ddagger$ $\quad (\Delta_{int}G/RT) = 21.46$

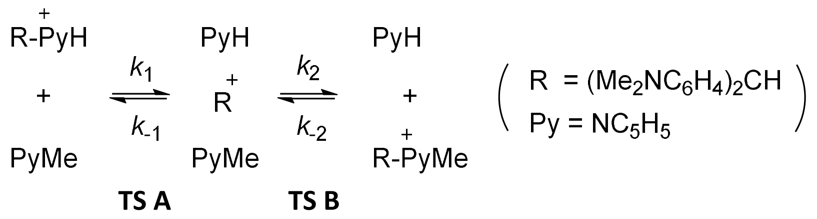

Scheme. S_N1 nucleophilic displacement.

The 2° α-D KIE for the overall reaction in the forward direction is given by eq 6, where A and B now refer to the TSs for the first and second steps in the Scheme, and the k_A and k_B values are both with respect to the common reactant state.

$$ {}^{\mathrm{D}}k_{\mathrm{app}} = w_{\mathrm{A}}\,{}^{\mathrm{D}}k_{\mathrm{A}} + w_{\mathrm{B}}\,{}^{\mathrm{D}}k_{\mathrm{B}} \qquad (6) $$

Table S5. Weighted average IPFRs and KIEs for completely rate-limiting step 1 or step 2.

species	$\langle \alpha\text{-}^2\mathrm{H}\, f \rangle$	rate-limiting KIE = f_{RS}/f_{TS}
$RPyH^+$	47.427159	
TS A	41.634706	1.139
TS B	41.150141	1.152

Table S6. Variation in Gibbs energy difference $(\Delta\Delta^{\ddagger}G/RT)_{\mathbf{rc}} = (G_B - G_A)$ as a function of the relative concentration **rc** = [PyMe]/[PyH], together with the consequent changes in weighting factors w_A and w_B for TSs A and B, the weighted average TS IPFR = $\langle \alpha\text{-}^2\mathrm{H}\, f^{\ddagger} \rangle$ and the weighted average KIE at 298 K.

[PyMe]/[PyH]	$(\Delta\Delta^{\ddagger}G/RT)_{\mathbf{rc}}$	$\log_{10}([\mathrm{PyMe}]_{\mathrm{rel}})$	n_{AB}	w_A	w_B	$\langle \alpha\text{-}^2\mathrm{H}\, f^{\ddagger} \rangle$	$\langle$ KIE $\rangle$
0.03	7.673	-1.523	2150	0.0005	0.9995	41.150366	1.1525
0.1	4.688	-1.0	108.7	0.0091	0.9909	41.154559	1.1524
0.3	1.965	-0.523	7.135	0.123	0.877	41.209707	1.1509
0.5	0.699	-0.301	2.011	0.332	0.668	41.311067	1.1480
0.7	-0.135	-0.155	0.8734	0.534	0.466	41.408803	1.1453
1	-1.020	0.0	0.3607	0.735	0.263	41.506246	1.1427
2	-2.738	0.301	0.06471	0.939	0.061	41.605257	1.1399
5	-5.009	0.699	0.006675	0.9934	0.0066	41.631493	1.1392
10	-6.728	1.0	0.001196	0.9988	0.0012	41.634127	1.1391
20	-8.446	1.301	0.000215	0.9998	0.0002	41.634602	1.1391

REFERENCES

(1) Matić, M.; Denegri, B. DFT-PCM study on solvolytic behaviour of *N*-alkyl-*X*-pyridinium ions. *ChemistrySelect* **2021**, *6*, 2410-2423.

(2) Gaussian 16, Revision C.01, Frisch, M. J.; Trucks, G. W.; Schlegel, H. B.; Scuseria, G. E.; Robb, M. A.; Cheeseman, J. R.; Scalmani, G.; Barone, V.; Petersson, G. A.; Nakatsuji, H.; Li, X.; Caricato, M.; Marenich, A. V.; Bloino, J.; Janesko, B. G.; Gomperts, R.; Mennucci, B.; Hratchian, H. P.; Ortiz, J. V.; Izmaylov, A. F.; Sonnenberg, J. L.; Williams-Young, D.; Ding, F.; Lipparini, F.; Egidi, F.; Goings, J.; Peng, B.; Petrone, A.; Henderson, T.; Ranasinghe, D.; Zakrzewski, V. G.; Gao, J.; Rega, N.; Zheng, G.; Liang, W.; Hada, M.; Ehara, M.; Toyota, K.; Fukuda, R.; Hasegawa, J.; Ishida, M.; Nakajima, T.; Honda, Y.; Kitao, O.; Nakai, H.; Vreven, T.; Throssell, K.; Montgomery, J. A., Jr.; Peralta, J. E.; Ogliaro, F.; Bearpark, M. J.; Heyd, J. J.; Brothers, E. N.; Kudin, K. N.; Staroverov, V. N.; Keith, T. A.; Kobayashi, R.; Normand, J.; Raghavachari, K.; Rendell, A. P.; Burant, J. C.; Iyengar, S. S.; Tomasi, J.; Cossi, M.; Millam, J. M.; Klene, M.; Adamo, C.; Cammi, R.; Ochterski, J. W.; Martin, R. L.; Morokuma, K.; Farkas, O.; Foresman, J. B.; Fox, D. J. Gaussian, Inc., Wallingford CT, 2016.

(3) Williams, I. H.; Wilson, P. B. SULISO: The Bath suite of vibrational characterization and isotope effect calculation software. *SoftwareX* **2017**, *6*, 1-6.

(4) Williams, I. H. On the Representation of Force Fields for Chemically Reacting Systems. *Chem. Phys. Lett*. **1982**, *88*, 462-466.

(5) Williams, I. H. Force-Constant Computations in Cartesian Coordinates. Elimination of Translational and Rotational Contributions. *J. Mol. Struct. THEOCHEM* **1983**, *11*, 275-284.

(6) Bell, R. P. The tunnel effect correction for parabolic potential barriers. *Trans. Faraday Soc.* **1959**, *55*, 1–4.

(7) Bell, R. P. *The Proton in Chemistry*, 2nd ed. Chapman and Hall: London, U.K. 1973.
